# Cutaneous Squamous Cell Carcinoma: From Diagnosis to Follow-Up

**DOI:** 10.3390/cancers16172960

**Published:** 2024-08-25

**Authors:** Rosita Comune, Angelo Ruggiero, Antonio Portarapillo, Alessia Villani, Matteo Megna, Stefania Tamburrini, Salvatore Masala, Giacomo Sica, Fabio Sandomenico, Chandra Bortolotto, Lorenzo Preda, Mariano Scaglione

**Affiliations:** 1 Department of Precision Medicine, University of Campania Luigi Vanvitelli, 80138 Naples, Italy; 2 Section of Dermatology, Department of Clinical Medicine and Surgery, University of Naples Federico II, 80131 Naples, Italyali.vil@hotmail.it (A.V.); mat24@libero.it (M.M.); 3 Department of Radiology, Ospedale del Mare-ASL NA1 Centro, 80147 Naples, Italy; tamburrinistefania@gmail.com; 4 Department of Medicine, Surgery and Pharmacy, University of Sassari, Piazza Università, 21, 07100 Sassari, Italy; samasala@uniss.it (S.M.); mscaglione@uniss.it (M.S.); 5 Department of Radiology, Monaldi Hospital, Azienda Ospedaliera dei Colli, 80131 Naples, Italy; giacomo.sica@ospedaledeicolli.it; 6 Radiology Unit, Buon Consiglio Fatebenefratelli Hospital, 80123 Naples, Italy; f.sandomenico@istitutotumori.na.it; 7 Diagnostic Imaging and Radiotherapy Unit, Department of Clinical, Surgical, Diagnostic, and Pediatric Sciences, University of Pavia, 27100 Pavia, Italy; chandra.bortolotto@unipv.it (C.B.); lorenzo.preda@unipv.it (L.P.); 8 Radiology Institute, Fondazione IRCCS Policlinico San Matteo, 27100 Pavia, Italy; 9 Department of Radiology, James Cook University Hospital, Marton Road, Middlesbrough TS4 3BW, UK

**Keywords:** cutaneous squamous cell carcinoma, imaging CT, metastasis, lung metastasis

## Abstract

**Simple Summary:**

Cutaneous squamous cell carcinoma is among the most frequent cutaneous tumors. Over the years, the understanding and diagnostic sensitivity has increased due to the introduction and improved knowledge of dermoscopy, as well as of more recent noninvasive technologies, such as reflectance confocal microscopy and line-field confocal optical coherence. Imaging plays a central role in the staging of the disease; however, its role in the follow-up, as well as the choice of the best technique in each patient, is still not fully clarified.

**Abstract:**

Cutaneous squamous cell carcinoma (SCC) is the second most frequent skin cancer, accounting for approximately 20% of all cutaneous malignancies, and with an increasing incidence due to the progressive increment of the average age of life. The diagnosis is usually firstly suspected based on clinical manifestations; however, dermoscopic features may improve diagnostic sensitivity in cases of an uncertain diagnosis and may guide the biopsy, which should be performed to histopathologically prove the tumor. New diagnostic strategies may improve the sensitivity of the cutaneous SCC, such as reflectance confocal microscopy and line-field confocal optical coherence, for which increasing data have been recently published. Imaging has a central role in the staging of the diseases, while its exact role, as well as the choice of the best techniques, during the follow-up are not fully clarified. The aim of this literature review is to describe diagnostic clinical and instrumental tools of cutaneous SCC, with an insight into the role of imaging in the diagnosis and follow-up of cutaneous SCC.

## 1. Introduction

Cutaneous squamous cell carcinoma (SCC) is the second most frequent skin cancer, accounting for approximately 20% of all cutaneous malignancies [1]. Its incidence is progressively increasing due to the progressive increase in average life expectancy [2].

Several risk factors have been linked to the onset of SCC. The chance of having SCC increases dramatically with age—growing older is one of the main risk factors [3]. The lower melanin levels in light skin types, especially those with pale complexions, freckles, and light-colored eyes or hair, put them at greater risk because melanin offers some protection against ultraviolet (UV) radiation. Studies reveal that men have greater incidence rates of SCC than women do, possibly as a result of differences in skin care routines and outdoor job exposures [4]. Male sex is another risk factor. Exposure to UV radiation, from artificial sources such as tanning beds as well as natural sunlight, is a known risk factor. UV radiation can cause direct DNA damage in skin cells, leading to mutations that drive the development of SCC. Individuals undergoing immunosuppressive therapies, such as organ transplant recipients, are at increased risk because their weakened immune systems are less capable of identifying and eliminating cancerous cells [4]. Human papillomavirus (HPV) infections, particularly with high-risk strains like HPV 16 and 18, have also been associated with SCC, especially in the anogenital region and oropharynx [5]. Smoking is another significant risk factor, as the carcinogens in tobacco can cause genetic mutations in epithelial cells [6]. Additionally, certain genetic syndromes, such as xeroderma pigmentosum and epidermodysplasia verruciformis, increase susceptibility to SCC due to inherent defects in DNA repair mechanisms or immune function [4]. Collectively, these risk factors underscore the multifactorial nature of SCC development and highlight the importance of preventive measures and early detection strategies.

The prognosis is strictly related to the stage of the disease, as in most cases of cutaneous SCC, it does not affect the patient’s prognosis, with surgical excision being usually the first line and curative treatment [7]. In other cases, such as advanced SCC and SCC involving areas that are not surgically treatable, the management may be complex and may even result in patient death. Advanced SCCs include both locally advanced SCC and metastatic SCC. Locally advanced SCC are tumors that have invaded deeper tissues (muscle, nerve, or bone), or that are particularly large and cannot be treated with surgery or radiotherapy. Metastatic SCCs are tumors that have spread from the primary site invading skin, lymph nodes, or other organs [3,8]. Management of advanced SCCs includes instrumental imaging examinations necessary to define the extension and the structures invaded by the tumor. Herein, we report a literature review, aiming to resume the diagnostic and therapeutic management of SCC, with a focus on the role of imaging in the diagnosis and follow-up of SCC.

## 2. Materials and Methods

A comprehensive review of the English language medical literature was performed using Pubmed.

The following research terms “squamous cell carcinoma”, “NMSC” AND “Dermoscopy”, “Reflectance confocal microscopy”, “LT-OCT” AND “NMSC”, “therapies” AND “NMSC”, and “follow-up” AND “NMSC” have been included.

The following types of articles were included in the review: systematic reviews, metanalyses, clinical trials (CT), real-life studies (RLS). Case series and case report were excluded. Only the English language articles were considered, while French, German, and Spanish language manuscripts were excluded. Data regarding imaging technique were evaluated considering only the most relevant literature (guidelines, consensus, systematic revies, and meta-analysis). This manuscript does not contain any studies with human participants or animals performed by any of the authors, since it is only based on previously conducted studies.

## 3. Epidemiology

SCC is a non-melanocytic skin cancer originating from keratinocytes. The average age of onset is about 70 years, being about two times more frequent in males. The main risk factor for the development of SCC is UV exposure [4]. The incidence of SCC is not completely known and there is considerable geographical variation. Literature data show that incidences increase with age, male sex, and lower latitude. Indeed, several data reported variable incidence rates in different regions due to different UV exposure. In Australia, the incidence rate of SCC is the highest reported, being estimated around 467 per 100,000 person-years. The Australian rates significantly vary among the population, firstly depending on phototype. Particularly, in white patients (Phototype I and II), with sun-sensitive skin, the incidence of SCC is rising [9,10]. In the United States, the incidence is lower, being estimated at about 262 per 100,000 person-years [11,12]. In Europe, the incidence is estimated at 77 per 100,000 person-years [7,9]. Data from the Swedish Cancer Registry reported an increased incidence found in individuals living at the same latitude, which resulted higher than those who are resident in coastal areas, where hours of sunshine are higher than inland areas [12].

Recent studies showed the predominant role of UV exposure over other environmental carcinogens, and this may explain geographical differences in the incidence of SCC [13,14]. UV exposure induces the creation of two main products: the cyclobutene-pyrimidine dimers (CPD) and 6-4 photoproducts (6-4PP). CPDs account for 75% of the photoproducts and are the main UV-induced mutation observed. In addition to direct genomic damage, UV exposure induces the development of reactive oxygen species (ROS), which can oxidize DNA and alter its structure. Particularly susceptible to oxidative damage is guanine with the formation of 8-oxoguane, which can alter DNA structure by disrupting transcription and replication processes. Furthermore, ROS could lead to single strand breaks [15]. SCC may arise de novo or from actinic keratoses (AK). AK is an intradermal proliferation of atypical keratinocytes following chronic UV exposure [16]. The transformation and rate of progression of AK to SCC is highly variable. It is estimated that between 0–0.075% of actinic keratoses progress to SCC per year, while there is a 0.53% risk of AK progressing to SCC per year in patients with previous SCC [17].

## 4. Diagnosis

The diagnosis of cutaneous SCC is primarily clinical, based on the visual and tactile assessment of the lesion by a healthcare professional. Definitive diagnosis is achieved histologically through biopsy or excision of the lesion, allowing for microscopic examination and confirmation of cancerous cells. In addition to traditional clinical examination and histopathology, several advanced diagnostic aids have emerged, enhancing the accuracy and early detection of non-melanoma skin cancers (NMSCs), including SCC [18].

### 4.1. Clinical and Dermoscopic Findings

Clinically, cutaneous SCC presents variably depending on the stage of the lesion.

#### 4.1.1. Actinic Keratosis

Actinic keratosis (AKs), which may be the precursor to SCC, presents clinically as an erythematous, rough, squamous sub-centimetric papule, macule, or plaque at the light-exposed areas [19,20]. Objective dermatological examination of AKs is enough to make a diagnosis, as AKs are more easily palpable than visible [20]. Dermoscopy of actinic keratoses shows the strawberry pattern characterized by the following dermoscopic signs: red pseudo-network, thin vessels around the follicles, keratotic plug within the follicle, and white-yellow scales [19,20,21,22].

#### 4.1.2. Bowen Disease

SCC in situ (also known as Bowen disease) presents clinically as an erythematous and rough patch or slightly elevated plaque; occasionally, it can be pigmented [23]. Non-pigmented Bowen disease’ dermoscopy is characterized by disorganized glomerular vessels, yellow-white scales, double-edge signs, and pigmented structures located at the periphery of the lesion [24,25,26]. Pigmented Bowen disease’ dermoscopy is characterized by peripheral grey/black dots, occasionally organized in a streak or leaf-like structure [25].

#### 4.1.3. Invasive SCC

**Figure 1:**
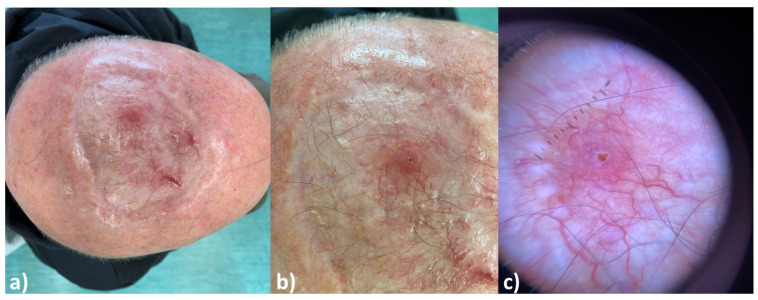
Typical clinical (**a**,**b**) and dermoscopic features (magnification 10×) (**c**) of cutaneous squamous cell carcinoma of the scalp in an elderly (82-year-old) male patient.

Invasive SCC (iSCC) clinically is a slightly elevated rough erythematous papule or plaque, frequently with a central ulceration [19,22]. There are several clinical subtypes, including keratoacanthoma and verrucous SCC. Keratoacanthoma could be considered as a highly differentiated form of cutaneous SCC, presenting as a rough, raised papule on an erythematous base with a large keratin plug localized at the center of the lesion [19]. Verrucous SCC presents clinically as an exuberant exophytic vegetative lesion [19,27]. iSCC in all its variants is asymptomatic but easily bleeds even after mild trauma [19]. The dermoscopy of iSCC is characterized by yellow-white keratin scales, white circles, structureless white areas, and ulceration. The vascular pattern is mixed and depends on the tumor differentiation grade. The vascular pattern includes linear vessels, hairpin vessels, and punctiform vessels. Larger irregular vessels and serpentine vessels are observed in undifferentiated iSCC [25,28,29] (Figure 1a–c).

### 4.2. Reflectance Confocal Microscopy

**Figure 2:**
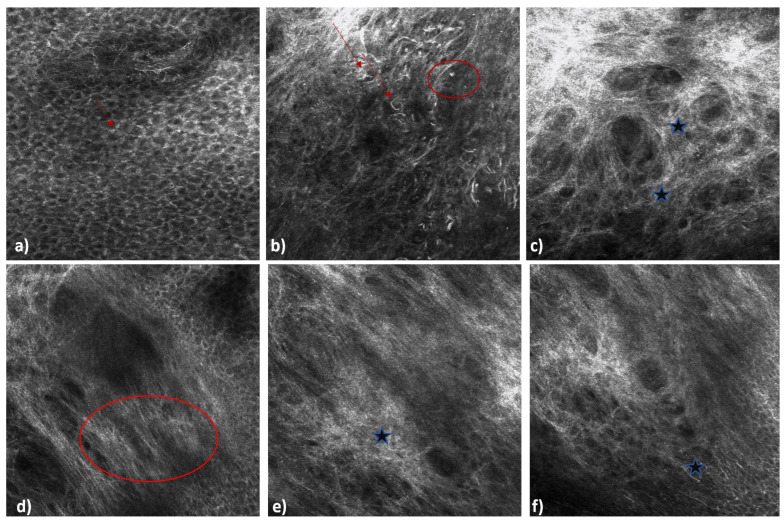
Reflectance confocal microscopy imaging of a cutaneous squamous cell carcinoma (VivaScope 1500/3000, MAVIG, GmBH, Munich, Germany, diode laser at wavelength of 830 nm). Rounded bright cells with hyperpigmented nuclei localized to the spinous and granular stratum organized in an irregular honeycomb pattern (**a**–**f**). Inflammatory cells are visible, as bright star-like dots (red arrows). Dilated vessels localized in the basal layer can be visualized (red circles).

Reflectance confocal microscopy (RCM) is an imaging technique that allows visualization of the epidermis and superficial dermis by providing in vivo and ex vivo histology-like images [30]. The basic principle of RCM is using a light source to assess skin lesions by the reflectance index of the structures examined. Although RCM is a new diagnostic method, it is widely used in dermatology, especially for skin tumors [30]. RCM in the diagnosis of AKs shows a thickened and irregular stratum corneum with keratinocytes with dark nuclei corresponding to orthokeratosis and parakeratosis. The spinous and granular stratum shows keratinocytes irregular in form and size with hyperpigmented nuclei forming a honeycomb structure [31]. Occasionally, inflammatory cells may be visible that appear on the RCM as bright star-like dots [31]. RCM of SCCs show rounded bright cells with hyperpigmented nuclei localized to the spinous and granular stratum organized in an irregular honeycomb pattern [32]. Dilated vessels localized in the basal layer can be visualized. The sensitivity and specificity of the RCM for the diagnosis of AK and SCC between 79–100% and 78–100%, respectively [30]. The main limitation of this method is the difficulty in differentiating AK, Bowen disease, and SCC, as these lesions share several histological features. Furthermore, pigmented AK and Bowen disease are not easily recognizable from other pigmented lesions especially from lentigo maligna often resulting in an overdiagnosis of melanoma [33] (Figure 2).

### 4.3. Line-Field Confocal Optical Coherence

Line-field confocal optical coherence (LC-OTC) is a new imaging technique that allows the study of the epidermis and dermis by acquiring horizontal, vertical, and three-dimensional (3D) images [34]. The advantages of this method are the possibility of studying the skin in depth while maintaining a high definition of the images comparable to RCM [34].

LC-OTC in the study of AKs allows the visualization of hyperkeratosis, pleomorphic keratinocytes with hyperpigmented nuclei and an intact dermo-epidermal junction (DEJ) [33]. Bowen’s disease on LC-OTC examination presents the same features as AK and differs mainly by a higher keratinocytes pleomorphism and by the presence of glomerular vessels [34,35]. The advantage of LC-OCT is the possibility to distinguish AK and Bowen disease from iSCC, which is characterized by interrupted or non-visible DEJ [36,37]. Although there are few data in the literature, LC-OTC is reported to be superior to RCM in the assessment of certain lesion characteristics such as dyskeratosis, parakeratosis, integrity of DEJ, and vascular pattern. Parakeratosis was observed in 70.6% at LC-OTC vs. 15% at RCM; dyskeratosis was observed in 82% at LC-OTC vs. 33.3% at RCM. Deeper lesions such as the vascular pattern, DEJ, and tumor strands are seen almost exclusively with LT-OTC [38].

### 4.4. Multiphoton Microscopy

Multiphoton microscopy (MPM) is advantageous for its ability to penetrate deeper into the tissue compared to traditional confocal microscopy [39]. MPM can highlight cellular and extracellular structures characteristic of SCC, such as increased cellular density and collagen remodeling [40]. Its application is mainly in research settings, but it holds potential for future clinical use due to its detailed imaging capabilities [39,40].

Each of these diagnostic modalities has unique advantages and limitations, and their comparative effectiveness varies depending on the clinical context. Dermoscopy and confocal microscopy are widely used in clinical practice due to their accessibility and high diagnostic accuracy. OCT provides valuable information on lesion depth and structure, making it useful for preoperative planning. Multiphoton microscopy, while currently more experimental, offers detailed molecular and structural insights that could enhance future diagnostic capabilities. Integrating these advanced techniques with traditional clinical and histopathological evaluation can improve the overall accuracy and efficiency of SCC diagnosis, ultimately leading to better patient outcomes.

## 5. Therapy

Treatment of SCC should be individualized based on the patient’s disease stage, location, and comorbidities. Surgical excision with a 4–6 mm margin of uninvolved skin is the first line of treatment for non-metastatic SCC ensuring resolution of the lesion. This approach ensures complete removal of the lesion and minimizes the risk of recurrence. Surgical excision is highly effective, with cure rates greater than 95% for primary SCCs. Major side effects include scarring and potential functional or cosmetic impairments, especially when lesions are located in cosmetically or functionally sensitive areas such as the face or hands.

Mohs surgery allows conservative excision to ensure tumor-free excision margins and is indicated for the treatment of SCC of difficult sites [19,41]. This technique allows for conservative tumor excision while ensuring clear margins, making it highly effective for the treatment of high-risk and recurrent SCCs. Mohs surgery offers cure rates of up to 99% for primary SCCs and approximately 94% for recurrent SCCs. Major side effects include prolonged procedural times and the need for specialized surgical skills.

Electrocoagulation, cryotherapy, and curettage are indicated for the treatment of small, low-risk SCC [19]. These methods are minimally invasive and effective for superficial lesions. However, they have lower cure rates than surgical excision, with recurrence rates of 5–10%. Potential side effects include scarring, changes in skin pigmentation, and, in the case of cryotherapy, blistering and pain at the treatment site.

In contrast, topical 5FU or imiquimod can be used to treat Bowen’s illness. Treatment options for several lesions in one area include topical 5FU, imiquimod, diclofenac 3%, and photodynamic therapy [19,42,43]. Imiquimod triggers the immune system to target cancer cells, whereas 5FU stops DNA synthesis in quickly proliferating cells. Patients who cannot have surgery or who have many injuries benefit most from these treatments. Localized skin reactions like redness, inflammation, and ulceration are examples of side effects.

A photosensitizing chemical is applied, and then a certain wavelength of light is exposed as part of photodynamic therapy. Actinic keratoses and Bowen disease are among the several superficial lesions for which this treatment is recommended. PDT is useful for producing nice cosmetic results with less scarring. Pain during the surgery, erythema following treatment, and photosensitivity reactions are common adverse effects. Radiotherapy is indicated for the treatment of high-risk inoperable SCC of difficult areas [43]. However, it is not indicated in young patients due to the risk of underdevelopment of other skin tumors in the treated sites [42]. It is also used as adjuvant therapy in cases where surgical margins are positive or in patients with perineural invasion. Radiotherapy is effective in controlling local disease but is associated with side effects such as skin atrophy, fibrosis, and an increased risk of secondary malignancies, particularly in younger patients. Oral prevention of NMSC represents a non-invasive treatment that guarantees a lower incidence of NMSC in high-risk patients. The only molecules indicated by international guidelines for the prevention of nmsc are retinoids and nicotinamide. Although they are the only molecules indicated, there is various evidence in the literature of molecules effective in the prevention of skin cancer such as polypodium leucotomos, beta-carotene, astaxanthin and celecoxib [44]. Prevention of sun damage with sunscreens and lifestyle modifications such as wearing hats and glasses, as well as early treatment of actinic keratosis, are effective preventive strategies to reduce the risk of the onset of nmsc [45].

## 6. Follow-Up

Squamous cell carcinoma occurs with extreme heterogeneity. The AIOM guidelines suggest a serious follow-up of high-risk SCC (diameter > 2 cm, deep infiltrating tumors, aggressive histological variant, perineural involvement, recurrent tumors, or involvement of difficult sites such as lip or ear) by lymph node ultrasound study every 3 months for the first 2 years, every 6 months for 3 years, and then once a year. There is extensive evidence of the usefulness of imaging examinations in the monitoring of SCC. Ruiz et al. showed in a study conducted in a population with T2B or T3 SCC that lymph node metastasis rates were higher in the population that did not perform instrumental staging examinations. Mortality was also higher in the group not performing staged imaging (32/53: 60.4%) than in the group performing staged imaging (19/45: 42.2%) [46,47]. Equally important is the dermatological follow-up of patients with SCC, as they are associated with an increased risk of developing other AK/SCC or other cutaneous malignancies [48,49].

## 7. The Role of Imaging in Cutaneous Squamous Cell Carcinoma

**Figure 3:**
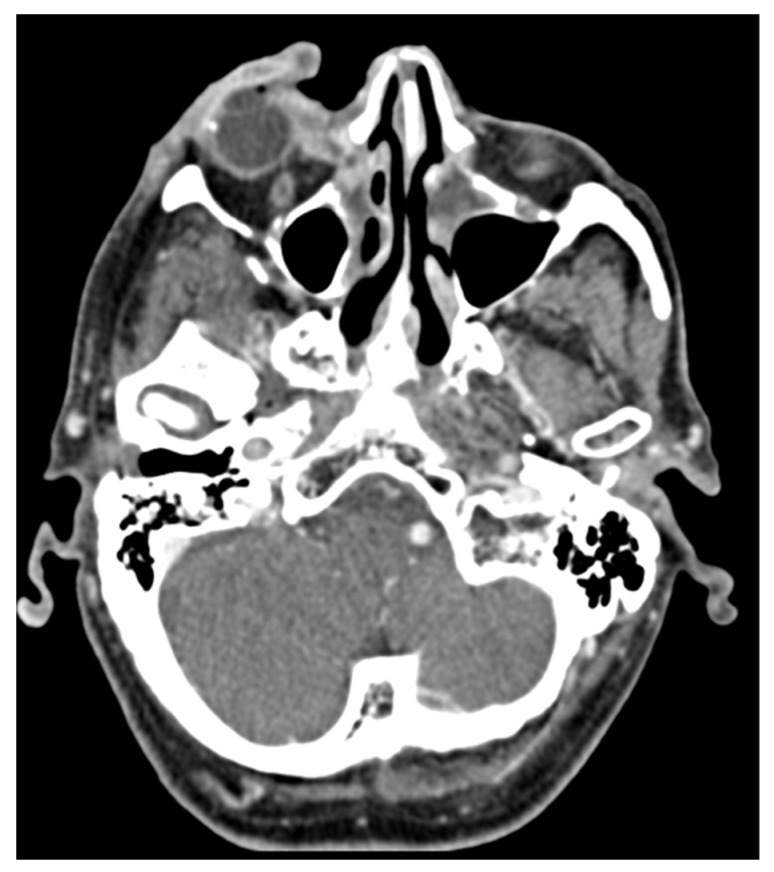
An 86-year-old male with squamous carcinoma of the right orbit, CT examination performed with administration of intravenous contrast shows the ulcerated skin and subcutaneous lesion in correspondence with the right orbit with peripheral enhancement with centrally necrotic-colliquative components. (Dual Source CT (Siemens Somatom), Siemens Healthcare, Forcheim, Germany).

To date, only a few data and minor evidence have been reported about the use and indication of imaging in cutaneous SCC. Indeed, both the choice of the most relevant technique and the correct indication of each one are still not fully clarified, with no studies evaluating each imaging in specific cutaneous SCCs, such as scalp SCC, high-risk, or advanced SCC [50,51]. As previously stated, the risk of lymph nodes metastasis is relatively low in cutaneous SCC. Thus, an indiscriminate imaging screening may lead to an increased number of false positives, and consequently non-necessary second level imaging, resulting in higher costs with no effective clinical benefits [52,53,54]. On the other hand, higher T scores (TNM classifications) have been associated with a significantly increased risk of lymph node involvement, suggesting the need of evaluating these patients at higher risk [5]. Indeed, it has been reported that the earlier identification of lymph nodes metastasis (with only few lymph nodes involved, and/or without extracapsular invasion), is associated with a better prognosis [55,56]. Indeed, some studies suggested to perform an ultrasound screening in patients with higher risk of lymph nodes involvement, leading the identification of lymph nodal metastasis at earlier stages than those performing imaging only in clinically detected cases [57]. Guidelines suggest that in cases of low-risk cutaneous SCC a detailed anamnesis and physical examination may be sufficient, while imaging should be reserved to clinically suspect cases [48,57,58]. In regards to high-risk cutaneous SCC, recent increased evidence suggests that even in case of negative physical examination, imaging may be able to identify subclinical stages of metastatic diseases, changing the therapeutical approach and significantly affecting the prognosis of patients [48,52,58,59,60]. A retrospective study showed that, even if linked to higher false positive rate, ultrasound examination had a significantly higher sensitivity in the detection of lymph node metastasis than physical examination alone [52]. Moreover, three retrospective studies evaluated the role of CT in cases of high-risk cutaneous SCC, showing that the using of CT imaging was linked to an earlier change in therapeutic approach, leading to better outcomes due to an earlier detection of more severe and invasive diseases [48,58,59]. Due to this increasing evidence, the staging approach changed over time. Indeed, the most recent European guidelines recommend performing imaging examinations (ultrasound examination and/or contrast-enhanced CT) in cases of high-risk cutaneous SCC, even in those with a negative clinical. The high-risk is defined by a T score of T2b or greater, or in the presence of the risk factors proposed by EADO [60]. In cases with clinical suspicious of bony invasion of the calvarium, it is crucial to evaluate the depth of tumor invasion. Contrast-enhanced CT may be preferred to ultrasound in cases of high-risk cutaneous SCC of the scalp, being able to assess both the lymph node involvement and the depth of the tumor, which may significantly differ from the clinically expected [59] (Figure 3). However, no official recommendations have been published about the specific scalp location. MRI is superior to CT in evaluating the deep invasion over the more external table of the skull, perineural invasion, parotid, or central nervous system involvement [61,62,63], Although contrast-enhanced CT has been proved to be superior to ultrasound, patients with high-risk forms, especially at the scalp, are frequently elderly patients, with an impairment of the kidney function, or kidney transplanted patients, conditions which may limit or constrain the application of contrast-enhanced CT. These may also be associated to a reduction in the sensitivity of contrast-enhanced CT. Thus, in these situations, ultrasound may be preferred to CT in the evaluation of lymph node involvement. Body CT and/or positron emission tomography (PET) should be performed in patients with a positive involvement of lymph node, to evaluate the presence of distant metastases [60].

**Figure 4:**
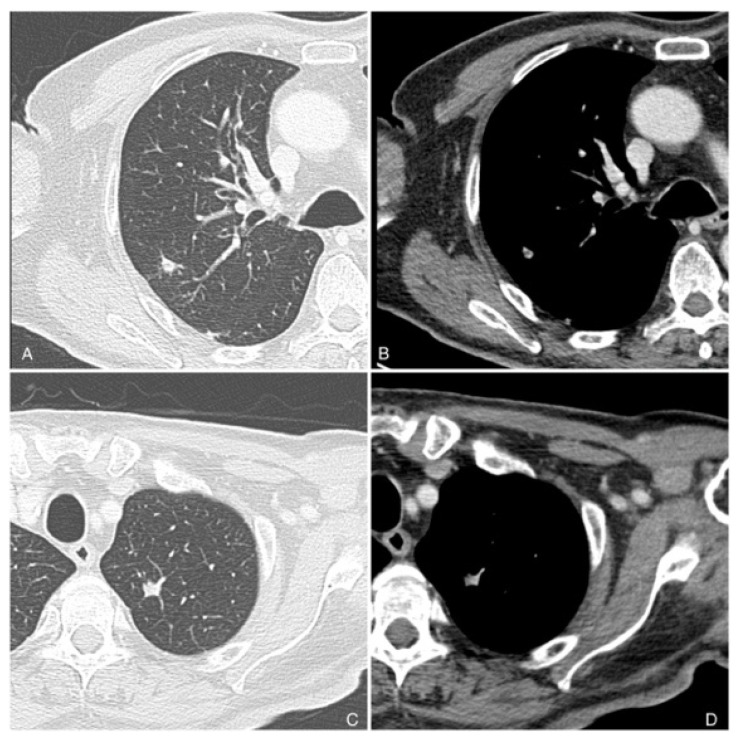
Chest CT images (**A**–**D**) of a 80-year-old male with squamous carcinoma of the internal canthus of the left orbit, shows two part-solid nodules, one found in the apical segment of the left upper lobe of the lung (**C**,**D**), and the other noted in the superior segment of the right upper lobe of the lung (**A**,**B**) were classified as intrapulmonary metastasis. Multidetector computer tomography of the abdomen and pelvis was performed with and without contrast. The examination was performed in the four-phase protocol, before and after contrast administration (Iomeron 370–400 Bracco, Milan, Italy, injected at 3.5/4 mL/s): unenhanced, arterial, portal venous and delayed phases, using a Dual Source CT (Siemens Somatom^®^, Siemens Healthcare, Forcheim, Germany).

High-risk cutaneous squamous cell carcinomas (SCCs) are associated with a significantly higher frequency of local recurrence and metastasis compared to low-risk SCCs, with recurrence rates reaching up to 35% [64]. The propensity for local and regional spread underscores the aggressive nature of high-risk SCCs. Regional lymph nodes, particularly those in the head and neck region, including the cervical and parotid lymph nodes, are the most frequently impacted sites of metastasis. These nodes are often involved due to the rich lymphatic drainage of the facial and scalp regions, which are common sites for high-risk SCCs [64]. The timeline for metastasis in SCC patients is critical for clinical management. Most metastases occur within two to three years following the initial diagnosis of SCC, highlighting the importance of vigilant follow-up during this period. Early detection of metastases can significantly influence the treatment approach and improve patient outcomes. In addition to regional lymph node involvement, high-risk SCCs have a notable potential for distant metastasis [64]. The most prevalent site of distant metastatic spread is the lung, followed by metastases to the brain, skin, and bone in about 15% of cases (Figure 4). Because of their effects on overall prognosis and respiratory function, lung metastases are particularly concerning [64]. Though less prevalent, brain metastases present several difficulties because of their impact on neurological function and the difficulty of treating them. Furthermore, significant morbidity, such as discomfort, pathological fractures, and a decreased quality of life, can result from metastases to the skin and bone. The frequency at these locations emphasizes the necessity of managing high-risk SCC with an all-encompassing, multidisciplinary strategy. Imaging modalities—such as positron emission tomography (PET), magnetic resonance imaging (MRI), and contrast-enhanced computed tomography (CET)—are essential for the early detection and tracking of metastatic disease. These instruments aid in determining the degree of disease dissemination and direct the selection of surgical, radiation, and systemic treatments, among other forms of treatment. While distant metastases, particularly those to the brain and lungs, are associated with a worse prognosis, intensive treatment is frequently beneficial in controlling local and regional metastases. The disparity in results emphasizes the necessity of ongoing investigations into better medicines and the creation of focused treatments to raise the survival rates of individuals with high-risk.

### 7.1. CTA Protocol

A CT scan is performed in patients using a multi-detector CT (MDCT) system with multiphase volumetric CT acquisitions. The patient is in the supine position (1.0 mm slice thickness with 0.625 mm reformations, 512 × 512 matrix and 40 × 40 cm FOV). A multiphasic CT is performed: non-contrast phase (to evaluate calcifications and baseline density) arterial phase (25–30 s after the second bolus for optimal arterial strengthening)venous phase (70–100 s after the initial bolus to capture venous structures and organs)delayed phase (5–10 min post-contrast for the detection of lesions in the liver and other organs). The arterial phase was obtained with the “bolus tracking” technique by positioning the region of interest (ROI) in the suprarenal abdominal aorta (value of 100 Hounsfield units, HU); The intravenous contrast medium is injected at a rate of 3.5–4 mL/s (approximately 100–130 mL depending on the patient’s weight), followed by a bolus of 20 mL of physiological solution at the same rate. CTs are reviewed using digital archiving and reporting software (Carestream PACS 11.0) on axial and multiplanar reforms (MPR).

### 7.2. US Protocol

In patients, an ultrasound is performed on the primary lesion and on one of the lymph node stations. For superficial structures a high frequency linear transducer (10–15 MHz) is used and for deeper lymph nodes a lower frequency transducer (5–7 MHz). The patient should be positioned comfortably, usually supine, depending on the location of the lesion and the regional lymph nodes to be examined. The location, size, and depth of the invasion should be documented and the echogenicity, margins, internal architecture, and involvement of adjacent structures of the lesion assessed. Measure the dimensions of the tumor in at least two perpendicular planes (length, width, and depth). We apply a color Doppler to evaluate the vascularity of the lesion, which can help distinguish between malignant and benign lesions. We perform a systematic examination of the regional lymph nodes and evaluate their size, shape, border definition, and internal architecture. Malignant lymph nodes often appear enlarged, with a round shape, loss of fatty hilum, and cortical thickening with abnormal vascularization. For cutaneous SCC of the head and neck, include the cervical, submandibular, and supraclavicular lymph nodes. For extremity lesions, include axillary or inguinal lymph nodes as appropriate.

## 8. Follow-Up Imaging

Regarding staging, the choice of clinical or imaging follow-up is mainly based on the type of SCC and risk factors. In patients with low-risk SCC, annual clinical follow-up, at least for the first two years, is the only recommended follow-up strategy. While follow-up by imaging is recommended in cases of high-risk cutaneous SCC, locally advanced disease, and metastatic disease [48,53,58]. For high-risk SCC, guidelines recommend performing an ultrasound of the lymph nodes (including the cervical and parotid lymph node area) every 3 to 6 months during the first two years [60,61,62,63,64,65,66]. This frequent monitoring is critical due to the increased likelihood of lymph node involvement in high-risk cases. The use of ultrasound is associated with excellent cost-effectiveness and should be preferred in patients in whom evaluation of the state of the skull is not required, such as in those with completely resected tumors [65]. However, some studies have reported that with ultrasound screening at baseline, only a few lymph node involvements are detected, whereas most are identified with clinical examination during follow-ups [57]. Furthermore, the common presence of comorbidities of renal blood chemistry alterations in these patients often increases the preference for ultrasound over contrast-enhanced CT, which may pose a risk of nephrotoxicity. However, some studies have reported that basic screening ultrasound detects only a few lymph node involvements, most of which are identified through clinical examination during follow-ups. This limitation suggests that the current timing of routine ultrasound may require adjustment to improve detection rates. Further studies are needed to evaluate the real efficacy and role of ultrasound, to clarify the optimal timing and sensitivity of this modality during follow-ups. In cases of locally advanced and metastatic cutaneous SCC, a follow-up frequency of 3–6 months is also recommended. The choice of imaging technique in these patients must be individualized based on clinical judgment. For these patients, CT examination may be preferable, especially to evaluate any brain lesions, detect bone involvement and monitor disease progression or response to therapy [60]. CT imaging is particularly beneficial in cases where there is suspicion of deeper tissue or bony involvement that ultrasound may not adequately visualize. This is especially relevant for high-risk SCCs of the scalp, where the assessment of potential calvarium invasion is critical. The comprehensive imaging provided by CT can help guide surgical planning and other therapeutic decisions.

**Figure 5:**
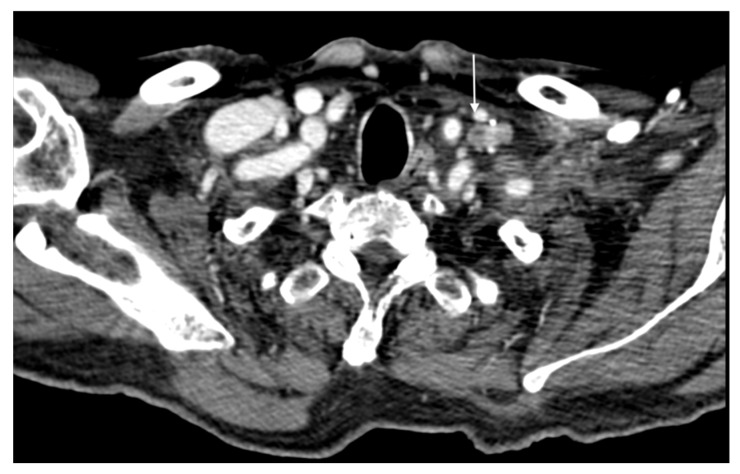
A 68-year-old man with known squamous cell carcinoma of the helix of the left auricle. Contrast-enhanced CT scan shows a positive latero-cervical lymph node. suspicious for metastatic involvement (white arrow). (Dual Source CT, Siemens Somatom^®^, Siemens Healthcare, Forcheim, Germany).

A consensus-based proposal for follow-up schedule for patients with history of cutaneous SCC has been proposed by several society, including, European Association of Dermato-Oncology (EADO), European Dermatology Forum (EDF), European Society for Radiotherapy and Oncology (ESTRO), European Organisation for Research and Treatment of Cancer (EORTC), and European Union of Medical Specialists (UEMS) (Table 1) [49]. However, the follow-up strategy for SCC should be tailored based on the risk level and specific clinical scenario. Low-risk SCCs require less intensive follow-up with clinical examinations, while high-risk, locally advanced, and metastatic cases benefit from regular imaging. Ultrasound offers an economical and safe option for lymph node evaluation, particularly in patients with renal comorbidities. In contrast, CT scans provide a more detailed evaluation needed for advanced disease management. However, studies evaluating the real efficacy and role of ultrasound are needed to better clarify the timing and sensitivity of ultrasound during follow-ups (Figure 5). Furthermore, studies should evaluate the long-term benefits of routine radiological examinations and optimize follow-up protocols to personalize patient care. Studying the integration of molecular and genetic profiling with imaging could lead to more precise and personalized treatment strategies.

## 9. Conclusions

One of the most common skin cancers is cutaneous squamous cell carcinoma (SCC). Its incidence is expected to increase in the coming years due to the constant increase in average age and life expectancy. Timely identification, diagnosis, and intervention are essential to improve patient outcomes. Although imaging is essential for determining metastatic disease and lymph node involvement, its function needs to be further clarified through new guidelines. Current guidelines lack specific recommendations on the best imaging modality, such as CT or ultrasound, in various clinical circumstances. Subsequent investigations should focus on evaluating individual justifications for different imaging modalities to fine-tune the decision between CT and ultrasound in a range of clinical settings. Studies should also evaluate the actual value of routine radiological examinations. These investigations would improve and customize long-term, follow-up techniques by assisting in identifying the imaging modality that works best for each patient and when to schedule radiological exams. The cost-effectiveness and possible hazards of various imaging modalities should also be investigated, particularly in patients with concomitant diseases such renal impairment that may restrict the use of contrast-enhanced CT. Examining non-conventional imaging modalities like MRIs or sophisticated ultrasound technologies like elastography and contrast-enhanced ultrasonography may offer important new perspectives on safer and more efficient imaging choices for high-risk individuals. The creation of non-invasive biomarkers that can support imaging investigations is a crucial topic for further study. These indicators may aid in the identification of individuals who are more likely to experience a recurrence or develop metastases, enabling more individualized and prompt therapies. When imaging is combined with genetic and molecular analysis, precision medicine approaches to the treatment of cutaneous SCC may become possible. Furthermore, longitudinal studies are required to evaluate the long-term effects of various follow-up tactics. The effects of various follow-up times and imaging modalities on patient survival, quality of life, and medical expenses ought to be investigated in these investigations. Determining the ideal frequency and length of imaging studies and follow-up visits is critical to creating evidence-based guidelines that strike a compromise between the requirement for comprehensive surveillance and the objective of reducing patient burden and healthcare costs.

Further studies should also consider the limitations of the available literature data, focusing on the potential variability in imaging results depending on patient demographics and disease stage. In summary, even though cutaneous SCC is still a major clinical problem, patient outcomes could be greatly enhanced by continued research and advancements in imaging methods and follow-up plans. The current gaps in knowledge and practice must be filled via collaborative efforts in clinical research, guideline development, and technological innovation. This will ultimately result in more effective and individualized therapy for patients with cutaneous SCC.

## Figures and Tables

**Table 1:** Follow-up schedule for patients with history of cutaneous SCC.

	Clinical Examination				Ultrasound of Lymph Nodes				CT, MRI, PET/CT			
Follow up time (years)	1	2	3–5	6+	1	2	3–5	6+	1	2	3	4+
Low-risk SCC	12 m	12 m	-	-	-	-	-	-	-	-	-	-
High-risk SCC	3–6 m	3–6 m	12 m	12 m	3–6 m	3–6 m	-	-	-	-	-	-
Locally advanced or metastatic SCC	3 m	3 m	3 m	6–12 m	3–6 m	3–6 m	3–6 m	6–12 m	3–6 m	3–6 m	3–6 m	Based on the response to treatment

CT, computed tomography; m, months; MRI, magnetic resonance imaging; PET, positron emission tomography.
